# Towards a Liquid Self: How Time, Geography, and Life Experiences Reshape the Biological Identity

**DOI:** 10.3389/fimmu.2014.00153

**Published:** 2014-04-09

**Authors:** Andrea Grignolio, Michele Mishto, Ana Maria Caetano Faria, Paolo Garagnani, Claudio Franceschi, Paolo Tieri

**Affiliations:** ^1^Interdepartmental Center “Luigi Galvani” for Bioinformatics, Biophysics and Biocomplexity, University of Bologna, Bologna, Italy; ^2^Centro Interdipartimentale di Ricerca sul Cancro “G. Prodi”, University of Bologna, Bologna, Italy; ^3^Institut für Biochemie, Charité – Universitätsmedizin Berlin, Berlin, Germany; ^4^Departamento de Bioquímica e Imunologia, Instituto de Ciências Biológicas, Universidade Federal de Minas Gerais, Belo Horizonte, Brazil; ^5^Department of Experimental, Diagnostic and Specialty Medicine, University of Bologna, Bologna, Italy; ^6^IRCCS of Neurological Science, Bologna, Italy; ^7^Institute of Organic Synthesis and Photoreactivity, National Research Council, Bologna, Italy; ^8^Institute for Applied Mathematics “M. Picone”, National Research Council, Rome, Italy

**Keywords:** self, non-self, antigen presentation, gut microbiota, host–pathogen interaction, *N*-glycan, oral tolerance, proteasome splicing

## Abstract

The conceptualization of immunological self is amongst the most important theories of modern biology, representing a sort of theoretical guideline for experimental immunologists, in order to understand how host constituents are ignored by the immune system (IS). A consistent advancement in this field has been represented by the danger/damage theory and its subsequent refinements, which at present represents the most comprehensive conceptualization of immunological self. Here, we present the new hypothesis of “liquid self,” which integrates and extends the danger/damage theory. The main novelty of the liquid self hypothesis lies in the full integration of the immune response mechanisms into the host body’s ecosystems, i.e., in adding the temporal, as well as the geographical/evolutionary and environmental, dimensions, which we suggested to call “immunological biography.” Our hypothesis takes into account the important biological changes occurring with time (age) in the IS (including immunosenescence and inflammaging), as well as changes in the organismal context related to nutrition, lifestyle, and geography (populations). We argue that such temporal and geographical dimensions impinge upon, and continuously reshape, the antigenicity of physical entities (molecules, cells, bacteria, viruses), making them switching between “self” and “non-self” states in a dynamical, “liquid” fashion. Particular attention is devoted to oral tolerance and gut microbiota, as well as to a new potential source of unexpected self epitopes produced by proteasome splicing. Finally, our framework allows the set up of a variety of testable predictions, the most straightforward suggesting that the immune responses to defined molecules representing potentials antigens will be quantitatively and qualitatively quite different according to the immuno-biographical background of the host.

## Introduction

In its historical development, the conceptualization of the immune self has always suffered of an “ontological obsession” (Box 1). Self had been focused on physical entities that Burnet identified as B lymphocytes (i.e., cells) (1, 2) and, later on, Janeway and Matzinger identified as pathogen-associated molecular patterns (PAMPs) (3, 4) and danger signals (endogenous, non-foreign damage/alarm signaling molecules) (5, 6), respectively (Box 2).

Box 1 **Rise, triumph, and decline of immune self.**

As an implicit concept, the notion of “biological individuality” raised in the second half of the nineteenth century (7). Bernard’s *milieu intérieure* (internal environment) as a prerequisite of life itself and Virchow’s cellular ability to preserve the identity against external attacks represent two examples of an auroral phase in which the dichotomy between the organisms’ individuality and their environment began to be introduced in the biological thought. Along these lines, Metchnikoff and Ehrlich gave a pivotal contribution in underpinning the “biological individuality” on specific immunological level, the former by proposing cell theory of phagocytosis as a pathogen-engulfing mechanism able to maintain organismal identity (7), the latter by suggesting the concept of “*horror autotoxicus*” to explain how immune system (IS) avoids toxic reactions against the endogenous constituents.

Seminal investigations were later on advanced by Loeb upon “the biological basis of individuality,” which in 1927 explicitly questioned the biochemical dynamics of graft rejection in different individuals (8), and in 1945 discussed individual specificity as a consequence of relationships among genes, enzymes, and blood groups, also including psychological and social dimension (9). However, the first scientist to put forth a purely immunological concept of selfhood, so as to become the theoretical backbone of the discipline, was the Australian immunologist Frank M. Burnet in 1949. Linked to the notion of “tolerance,” the “immunological self” initially advanced by Burnet coincided with all cell surface pattern recognitions that are ignored by the normal immune defensive action, namely the biological constituents peculiar to each individual (2). With the beginning of the 1960s, the notion of self became the pivotal concept of Burnet’s clonal selection theory, a new model which states that a randomly diversified population of clones – and not, as previously thought, a single adaptable antibody – was responsible of selecting the antigen, put the entire immunology on the right Darwinian track after almost half a century of Lamarckian approaches. The SNS paradigm was indeed coeval with the entry of immunology in the mainstream of the dominant Darwinian disciplines like genetics and paleontology involved in the Modern Evolutionary Synthesis, a conceptual frame that led many dispersed biological fields to a common selective paradigm during the 1930s and 1940s. In the subsequent two decades (1960–1980), the theory of “self–non-self” (SNS) has enjoyed a large and growing credit amongst immunologists, imparting a new impetus to the entire discipline.

Also, it offered an immediate theoretical framework to novel outcomes such as the idea of “immunological surveillance” of tumors, the early experimental successes of tissue transplantation tolerance, and the discovery that T cell recognition depended on the restriction of major histocompatibility complex (MHC) coded by human leukocyte antigen (HLA) genes (10).

In the early 1980s, several experimental data confirmed that the IS may not only sometimes fail to mount the expected immune response against foreign material (non-self elements), but, as in the case of autoimmune diseases, it also directs its activity against autologous materials (self elements). In this new scenario, SNS discrimination began to be seen as a useless or erroneous theory and its progressive debunking can be summarized in three phases.

*Phase I*: Represented by isolated attempts, the first phase coincided with the discovery of “clonal anergy,” which demonstrated that in immature mammals some self-reactive B cells might survive in a dormant (anergic) state, potentially becoming clones tolerant toward non-self elements in adulthood (11). Further criticisms arrived from the reappraisal of induced oral tolerance, which challenged the idea of a *stable* immune self in mature mammals (12). An offensive also mounted on a pure theoretical level, when in the mid 1970s, the influential Niels Jerne’s idiotypic network theory first suggested a selfless hypothesis to explain the whole immune activity: any concept of foreignness (and of selfhood) was in fact excluded in this theory, which stated that any antibody might be directed to either external antigens or body’s constituents.

*Phase II*: A second phase arrived with the post-self “costimulatory” models, which attempted to reconcile SNS theory with the chronic production of auto-reactive molecules. Proposed between the 1970s and the 1980s, the first step was took by Bretscher and Cohn’s two-signal model, which described the necessity of a “help” signal and a cellular co-stimulation to induce the mechanism of SNS discrimination in the elimination of self-reactive B cells (13). Inspired by this first attempt, Lafferty and Cunningham’s two-signal model further introduced a new “accessory cell,” i.e., the antigen-presenting cell (APC) and a new costimulatory signal to support T helper (Th) lymphocytes in evaluating the actual foreignness of the antigenic molecules (14); Janeway’s infectious-non-self model finally suggested that in the antigen recognition process some “costimulatory cells,” endowed with a quiescent ability to distinguish between “infectious non-self” and “non-infectious self,” were triggered by a chain of signals whereby a set of germ line-encoded pattern recognition receptors (PRRs) recognizes conserved PAMPs on bacteria (3, 4).

*Phase III*: A third phase began at the turn of the 1990s with the proposal by Coutinho, Cohen, Cunliffe, Dembic, and Matzinger of the post-self theoretical models. As a main proponent of the “second generation” of the Jernian network theorists (also called Paris School) (15), Coutinho advanced the idea that autoimmunity should be considered a normal physiological function – an “active-resting” state detectable with general patterns of collective, low-titer (auto-)antibody reactivities (16, 17) – being the IS activity twofold: a central network devoted to regulate itself with itself, collecting auto-reactive and connected lymphocytes and serving as a host-monitoring system, as well as a peripheral network made by disconnected clones, bereft of auto-reactive cells, ready to be triggered by foreign antigen (18, 19). With the hypothesis of “immunological homunculus,” also Cohen integrated, insomuch as to become a physiological function, the autoimmune response (20, 21). Here, rather than replaced, the Burnettian theory of self is overturned for the autoimmunity and the constant surveillance of non-self elements becomes a regular activity of a self-monitoring IS to maintain an internal equilibrium. The idea to abandon the mechanism of self–non-self discrimination in favor of a “morphostasis” process governing the tissues’ disorganization (cytokines-burst) was advanced by Cunliffe (22) and then significantly re-elaborated by Dembic (23, 24) with the “integrity hypothesis,” which proposes three groups of signals coordinating immunocytes and dendritic cells actions. As the most critical immunologist against the SNS theory, Matzinger’s danger model suggested that to activate an appropriate immune response, the APCs need to be co-stimulated by “endogenous, non-foreign danger/alarm signals,” provided by the surrounding environment in presence of stressed, damaged, or infected cells (5). This model specifically suggested a cellular process in which the discriminatory concept of “foreignness” of the constituents is no longer the condition for the activation of the immune response (e.g., mother/fetus relationship), as well as “immunological identity” of the host tissue is no longer a guarantee of tolerance (e.g., cell mutation or cancer).

Notwithstanding its originality, which mainly consists in reorienting the activity of IS as concerned with entities that do damage rather than with those that are foreign, the danger model appears to be still focused on a materialistic view and a undefined temporal dimension. On one hand, Matzinger’s theory, in a way similar to costimulatory models, points to the materiality of signals, just suggesting a shift from non-self signals (emanated by “anything that is foreign or new”) to danger/damage signals (emanated by “anything that induces stress or non-physiological death of a cell”). Along these lines, the danger model improves the classic discriminatory concept of aggression/tolerance by adding further “endogenous” signals such as pathogens, environmental toxins or mutations that lead to stress or inappropriate cell death or inefficient scavenging. On the other, such a theory offers no insight on geographical and population contexts, offering only a brief look at the different ontogenetic phases of the host. In this theory, as well as in all the previous ones, the temporal/biographical dimension is unsatisfactorily represented (25). The various parameters that will change with time should be, on the contrary, considered inevitable and intrinsic in the IS dynamics.

From danger model back to Burnet conceptualization, all the variants of immunological self advanced insofar have been focused on “materialistic” (cellular or molecular) signals able to trigger the immune response. We think that a step forward could be taken by also considering the “procedural” (time and space-related) aspect, rather than a materialistic one, of the immune response.

Box 2 **What is self?**

What really is the immunological self? According to Burnet, who introduced the term in 1949, the immunological self coincided with all the cell surface pattern recognitions that are ignored by the normal immune defensive action, namely the biological constituents peculiar to each individual. Since Burnet, there have been roughly a dozen different attempts to find an answer, but none has found a general acceptance. They can be grouped in six major answers as follows.

The self is:

(1) everything encoded by the genome (26, 27);
(2) any tissue under the skin accessible to lymphocytes, including structures encoded by commensal genomes or excluding immune “privileged” sites such as brain, cornea, and testes (5);
(3) the set of peptides complexed with the MHC (28);
(4) specifics like APCs and thymic epithelium (29) or soluble molecules of B lymphocytes (20);
(5) a set of bodily proteins that exist above a certain concentration (30);
(6) the immune network itself, variously conceived (31, 32).

While these versions may be situated along a continuum between a severe genetic reductionism and complex organismal constructions (33), each shares an unsettled relationship to Burnet’s original dichotomous model of self and other (34).

Today, no longer is the identity of the host organism given or assumed, and, indeed, the definition of immune selfhood (35) embraces at least three diverse definitions:

(1) the “organismal self” – i.e., the epistemological functional category immunologists typically employ;
(2) the “immunological self” – an ontological construction that draws from molecular definitions and builds upon Burnet’s theory of tolerance;
(3) the “immune self” – a metaphysical formulation of the system-as-a-whole.

Reformulated after Ref. (5, 7, 35).

While playing a fundamental role in the development of the immunological thinking, and because of its lack of sufficient explanatory power of emergent phenomena such as inflammatory and autoimmune diseases (36), the concept of immunological self has nowadays revealed its main limits in excluding the host’s spatial (i.e., geographical and environmental) and temporal (phylo- and ontogenetic) dimensions. Indeed, according to the currently recognized theories, the IS seems to be suspended in a limbo without time and space. Our hypothesis here aims to instantiate that the immune responses triggering is also intimately linked to host’s spatial and temporal dimensions, which we will mainly refer to as the so-called host’s *immunological biography* (37).

Even the most modern conceptualization about immune response triggering, and namely the Matzinger’s danger theory (5, 6), shows problematic issues in several key points. In this particular aspect, other authors argue that the concept of “danger” is a theoretical suggestion, while, conversely, the idea of molecular “damage” signals has led to a number of experimental studies especially focused on endogenous damage, which is where the innovation of the danger theory lies (38). Several drawbacks (e.g., insufficient explanations of innate immunity and response to symbiotic bacteria, among others) afflict the danger theory that may finally prove to be not completely satisfying (36).

To overcome such limitations, here we propose a wider framework for the immune response triggering in which we hypothesize that: (1) the (definition of the) self is a *process*, an evolving configuration of states, and should *not* be strictly referred to a *physical entity* (ontology); (2) as a process, the self is dynamic because it varies depending on the internal (inflammatory, mostly) and external (ecological) contexts; (3) the self is mainly defined within a *continuum* of states by the immunological history at the species (evolutionary) and the individual (ontogenetic) level, i.e., by the *quantitative, qualitative*, and *temporal* aspects of the immunological stimuli that each of us is exposed to in our lifelong history. In particular, besides structure, dose, time, and localization of antigen (39), we underline the importance of the host’s temporal dimension in terms of antigen exposure *in utero* (40), during birth (41), and in senescence (42).

As a consequence, the old question whether a given molecule belongs to self or non-self looses its significance as it largely depends on the context (43), which will be here referred to as the wider perspective of the immunological biography (37).

Accordingly, by recovering the widely used sociologic metaphor advanced by Zigmunt Bauman to indicate amorphous, elusive, and rapidly changing values of contemporary Western societies (44, 45), we propose to make the final step from an ontological and static idea of self to a*, context-, spatial-, temporal-, inflammatory-, and finally process-dependent concept*, and thus introduce the hypothesis of *liquid self*.

We will discuss our hypothesis by testing it on some of the most forefront and fundamental immunological research areas, i.e., the gut microbiota–host interactions (see “Host–Pathogen Interaction and the Evolutionary Self”), the ubiquity of damage-associated molecular patterns (DAMPs) (see “DAMPs: When The Self Shifts Toward Non-Self”), the continuous antigenic assimilation in oral tolerance (see “Gut Microbiota–Host Interactions and Oral Tolerance: When the Non-Self Becomes Self”), the overlapping between pathogenic bacterial peptides and human peptides (molecular mimicry) (see “Molecular Mimicry and Proteasome-Catalyzed Peptide Splicing: When Self and Non-Self Coincide”) and the epigenetic modifications and mechanism of glycosylation in creating antigens signals (see “Age-Associated *N*-Glycans Modifications and Inflammation: When the Aged Self Becomes Non-Self”).

Finally, we will argue that the immunological self is at the intersection of long-term, distant, evolutionary selective force operating at the level of population and proximate immunological experiences at the level of individual’s lifetime. Genetic and immunological evidences indicate that the IS is molded and shaped by evolutionary forces where microbial infections played a major selective pressure, and whose final consequences are evident at the population level. On this evolutionary background – that can differ from population to population on the planet according to different environmental and epidemiological agents (46) – a successive remodeling of the IS occurs progressively with age, mainly driven by the individual-specific *immunological biography* (i.e., the sigma of the immune stimuli that a single individual has received lifelong) from utero to the last decades of life, in different environments, and related to quality and quantity of pathogens an individual has been exposed to. In conclusion, we will highlight how the concept of liquid self is well in accordance with recently proposed continuity/discontinuity hypothesis (47, 48), which recovers the idea that abrupt changes in the amount of antigen over time are critical in triggering immunity, a concept originally proposed by Grossman and Paul (49) and now recently expanded (50).

## Host–Pathogen Interaction and the Evolutionary Self

The host–pathogen interaction phenomena have profoundly shaped the genetic evolution of our species. One of the most paradigmatic examples is represented by the prevalence of thalassemia in Mediterranean regions, as a result of the selective pressure of malaria infection (51), the latter a disease that in this case plays a defensive role by contrasting through a natural selection mechanism, the more severe hemoglobin disorder (51, 52). While the malaria-inducing plasmodium has produced a clear mark of its presence, other pathogens may have left signatures that are more difficult to highlight, and probably many other alleles are present in the population’s gene pools as a result of adaptation to specific chronic inflammatory pressure (53). Genes involved in immunological processes are more exposed to the selective pressure exerted by pathogens. Indeed, comparative genomic studies have clearly shown that such genes are less conserved and frequently targeted by positive selection than genes involved in other pathways (54, 55). The evolutionary dynamic nature of the IS is also described by population genetics observations. The Wellcome Trust Case Control Consortium studies, where several thousands of individuals were genotyped by genome-wide association studies, reported that the genomic regions showing highest levels of population sub-structure (i.e., that are more differentiated between populations, as a result of a higher evolutionary rate) are the major histocompatibility complex (MHC) and the genes coding for toll-like receptor 1 (TLR1) (56). The MHC is one of the best-known examples of balancing selection in humans, and its extremely high level of heterozygosity is maintained by pathogen-driven selective pressure (57). The TLR1/TLR6/TLR10 region on chromosome 4p14 is characterized by high levels of heterozygosity and high levels of non-synonymous mutations (i.e., altering the amino acid sequence of a protein) with minimum allele frequency (i.e., >0.10 in Caucasian populations), with clear marks of positive/balancing selection. The explanation of such selective pressure is that TLR1 receptor, in a heterodimeric combination with TLR2, recognizes lipopeptides from Mycobacteria, the causes of leprosy and tuberculosis (58). These observations indicate that genetic diversity allows for increased flexibility in the immune response that is always struggling to reach an effective equilibrium between fighting invaders and the tolerance of self and of innocuous antigens (59).

In addition, some structural proteins (antigens) expressed by pathogens such as mycobacteria have a high homology with mammalian proteins, good examples of these are heat shock proteins (HSPs). HSPs are highly conserved proteins during evolution. Besides being upregulated in stress conditions, they are constitutively and abundantly present in all living beings and are profoundly involved in various intracellular and systemic homeostatic functions in different species (60, 61). These proteins have been considered by Irun Cohen (62) as immunodominant antigens for self immune responses and as key players in physiological autoimmunity, likely to be involved in homeostasis (63). In line with this idea, self-HSP-reactive T and B cell clones can be seen as an important part of a network of regulatory cells and molecules in the IS engaged in homeostatic activities (64). These activities would include tissue maintenance and repair, but also limiting clonal expansion and controlling inflammation. Interestingly, anti-HSP antibodies and HSP60-reactive T cells are part of immune responses in several infectious diseases (65). Approximately 10–20% of the specific T cells in mice immunized with *Mycobacterium tuberculosis* are against the bacterial HSP65 (66). Antibodies to HSP60 of Chlamydia trachomatis have been detected at high levels in the sera of infected patients (67), and immunodominant responses to HSP60 are present in other fungal infections (68). This strong immune response directed to HSP60 during infection can be explained by its critical role in cellular homeostasis and by its upregulation in host tissues as a result of stress during infection. According to Cohen, response to such homologous antigens would have a key role in connecting the immune response to pathogens to an internal web of regulatory circuits [mostly of regulatory T cells (T_reg_)] that could regulate specific immunity as well. These regulatory cells would control inflammatory responses to pathogens keeping them from damaging body tissues (62). Indeed, most of the pathological aspects associated with chronic infections are associated with the inflammatory responses that the infectious agents trigger in the host.

Taken together, all these clues strongly support the hypothesis that the host–pathogen interaction is the most crucial challenge *Homo sapiens* has faced during evolution. This interaction has led to the development of a vast arsenal of sophisticated weapons to fight a vast repertoire of pathogens, and vice versa to a variety of strategies that pathogens have evolved to evade the immune response. The drastic reduction of potential pathogens repertoire that we were usually exposed to in the (recent) past, particularly in the last century in western countries, has created a mismatch between these new environments and the one toward which the IS was adapted along the course of its evolution. While in the developing countries infectious diseases account for about 48% of deaths among people with 45 years or less of age (69), in the industrialized world the radical changes of the human environment and the development of efficient healthcare systems have resulted in a drastic reduction in the diversity/quantity of microbial species we usually deal with (70). Paradoxically, despite being a major and well recognized contributor to the recent increase of the lifespan in western countries, this consistent reduction of IS stimuli/antigens has in turn favored the development of chronic inflammatory conditions and autoimmune diseases (71), according to the so-called hygiene hypothesis (72). Specifically, quantitative and qualitative alterations of T regulatory (T_reg_) lymphocytes, key players in modulating immune reactivity and inflammation, imply an alteration of the inflammation homeostasis, leading toward autoimmune reactions (73). The human IS has co-evolved with many pathogens, including intestinal saprophytes and some helminths, against which the immune response is switched off (74). Many receptors, including TLRs, expressed on immature dendritic cells, are stimulated by antigens shown by the tolerated saprophytes and helminths, and this interaction stimulates the maturation of dendritic cells that in turn promote the T_reg_ cells-mediated responses to these organisms. This mechanism is a key regulator of the basal inflammatory tone and of the homeostasis of the immune response. By having reduced the number of these antigenic interactions, the new hygienic condition in industrialized countries has consequently reduced the stimulation of T_reg_ cells: a process that may lead to a disruption of the homeostasis and to the alterations of the basal inflammatory tone, thereby opening the door to an increasing incidence of autoimmune pathologies (59). In this complex stage, and on a wider scale, the human geographical dimension seems to play a consistent role. It has been observed that tropical Africans are specialized at fighting parasites with low virulence and extended life spans, while Europeans and perhaps Asians are better adapted to high virulence pathogens (75), suggesting that evolved responses to diverse pathogen loads among geographic groups may contribute to higher frequencies of inflammatory diseases in contemporary communities.

As pointed out by Ward and Rosenthal, TLR diversity is wider in echinoderms but smaller in mammals (76). Such TLR diversity goes in parallel with a multiplication of cytokine receptors and of the STAT adaptor proteins. This framework suggests that with the initiation of adaptive immunity, the abundance of innate immune receptors was selectively reduced, and the role of PRRs shifted to initiators of downstream signaling for the adaptive immunity (76). Organisms that developed a costly, complex, and specific system for fighting infection maintained the core of innate IS with the ability to activate it in an effective way.

Therefore, it is possible to speculate that the large number of sea urchins’ TLRs and the less TLRs in mammalian species are themselves a documentation of fluidity in the innate and adaptive world that represents our convoluted and highly evolved “self,” and that hard-wired recognition of “non-self” was too “dangerous” for continuity with an adaptive immune response in hand. An excessive number of “rough” recognizing events would have likely been dangerous and costly in this new “adaptive” situation. Indeed, there are additional evidences that evolutionary history of European populations has identified several immune pathways, and in particular TLR1/TLR6/TLR10-related pathways, as being shaped by convergent evolutionary pressure in two human populations with different origins under the same infectious environment, taking into account both their geographical and immuno-biographical characteristics (77).

Moreover, other types of immunologically important proteins, such as the five cytosolic toll/IL-1 receptor (TIR) adaptor proteins, now increasingly recognized to play a crucial role in the specificity of the response, evolved in humans by a swinging game between constraint pressure and adaptation (78), i.e., what, at the population level, we can consider equivalent to a “dirty” or a “clean” environment. Indeed, studies on human populations of host defense genes (with parallel roles in model organisms) have shown divergent evolutionary paths among the distinct receptor and adaptor proteins of the innate IS. These data are compatible with the hypothesis that natural selection exerts a strong effect on IS genes as a consequence of host adaptation to novel, diverse, and coevolving pathogens. In particular, the data suggest that the contemporary diversity of the five TIR adaptors arises from multiple events related to the pressure of specific constraints plus adaptive evolution, resulting in a dynamic divergence in time and space, even stronger than that observed on TLRs (78).

As an immuno-biographical example, we reported that in human subjects with very long and diverse immunological experience (nonagenarians and centenarians) the “immunological space” is filled with expanded clones of T cells specific for few epitopes of common and persistent viruses such as cytomegalovirus (CMV) and Epstein–Barr virus (EBV) (79, 80), concomitantly with a remarkable shrinkage of CD4+ and CD8+ T cell repertoire (81). Such adaptive outcomes can be considered a good example of how large the changes occurring in the adaptive IS with time/age/immunological experience can be. In a previous theoretical contribution (82), we argued that a significant amount of “degeneracy,” defined as the ability of structurally different elements to perform the same function (83), is required by the inherent complexity of IS in order to operate effectively, and that such degeneracy of T cells, as well as of other IS sensors (84, 85), likely represents a most important structural basis of the liquidity of the immune self. Most importantly, degeneracy is a prominent property of evolution itself and it has been primarily proposed as a evolutionary strategy: degenerate structures, indeed, are functionally overlapping and rearrange their configuration to meet internal or external (environmental) changes thanks to their interchangeable task capabilities, something that makes them capable of yielding unforeseen functionalities, and may thus show evolutionary advantage (83). Along these lines, we see the recent degenerate mechanism of the liquidity of self as a proximate cause of a long running evolutionary strategy of adaptation.

Finally, we surmise that the liquidity of self, possibly present at low level even in invertebrates, likely “exploded” with the appearance of adaptive immunity and its unprecedented capability to generate large T and B repertoires. A more adaptable and flexible immunity was carrying in itself the hallmarks of a modular detection of SNS, able to transform upon space and time changes. In the new evolved self also innate immunity plays a role in keeping self liquid (TLR1/6/10 genetic evolution in human population) but our theory of liquid self describes the IS and SNS detection of an organism, which has a predominant highly differentiated and modular adaptive immunity. Adaptive immunity and liquid self walked through the gate of evolution side by side.

## DAMPs: When the Self Shifts toward Non-Self

Damage-associated molecular patterns (also known as alarmins) are molecules released by injured cells undergoing necrosis and apoptosis (86) that act as endogenous danger signals able to promote inflammatory responses. Increased serum levels of DAMPs have been associated with several inflammatory diseases, including arthritis, atherosclerosis, lupus, Crohn’s disease, and cancer. DAMPs are perceived by the innate IS by PRRs that are also able to sense PAMPs. Recently, evidences of DAMPs resulting from nucleus, endoplasmic reticulum (ER), cytosol, and plasma membrane, as well as mitochondria-derived DAMPs (mito-DAMPs) have been reported. In particular, mito-DAMPs seem to occupy a central position as modulators of inflammation during different pathologies that are accompanied by massive cell death and/or tissue damage (87, 88) (e.g., 95% of patients with primary biliary cirrhosis have high titers of anti-mitochondrial antibodies (89), confirming that the dominant auto-reactive response is mounted against the 70 kDa mitochondrial antigen). It has been also observed that circulating mtDNA increases with age, and can significantly contribute to the maintenance of the low-grade, chronic inflammation (inflammaging) observed in elderly people (90). Such considerations suggest that mito-DAMPs, and related immune pathways, may be among the causes, hence possible targets, for the treatment of autoimmune and autoinflammatory diseases.

Metabolites such as nucleotides (ATP) or nucleosides (adenosines) once gained the extracellular milieu after cellular disruption act as danger signal and alert immune responses (91), although danger signaling is not only due to misplacement of such metabolites but also to their quantity. Indeed, the role of ATP could shift from up regulation of costimulatory molecules turning on inflammation at low concentration, to the blockade of the synthesis of the pro-inflammatory cytokines at higher concentration (92–94).

In conclusion, being endogenous and ubiquitous, mito-DAMPs would be unaccountable according to a substantially static SNS (ontogenetic) dichotomy. Taking into account that mtDNA has an ancestral bacterial origin, its capability to become a strong inflammatory stimulus when released outside the cell can be taken as a paradigmatic example that self DAMPs can trigger innate immunity receptors shifting toward non-self. HSPs, HMGB1, and fibrinogen can be taken as further examples of self molecules showing such “borderline” capability of activating TLRs (95, 96). The steady increase with age of circulating mtDNA reported in Ref. (90) also suggests that there is a continuum from young age to nonagenarians and centenarians regarding the possible role of mito-DAMPs to became detrimental and contribute to inflammaging, assumed as the background of major age-related diseases. When such age-related inflammation starts becoming detrimental, the entire scenario of DAMPs as self or non-self also starts to become quite blurred. In this framework, it is difficult or even impossible to trace the initial/original cause of the entire, dynamic inflammaging process, which is dominated by a series of vicious circles implementing and amplifying each other.

## Gut Microbiota–Host Interactions and Oral Tolerance: When the Non-Self Becomes Self

The intestinal mucosa lodges the largest lymphoid tissue. The gut-associated lymphoid tissue (GALT) contains a number of plasma cells and lymphocytes exceeding the number found in the other lymphoid organs altogether (97). This large lymphocyte-rich surface contacts daily an equally large collection of natural antigens coming from two sources: gut microbiota and diet. It is estimated that we eat 190 g of protein antigens daily and that the number of bacteria colonizing the human intestine is around 10^12^ bacteria/g of stool (98). Thus, the gut microbiota provides a variety of antigenic components continuously, and a significant part of the dietary proteins reaches the circulation in its intact immunogenic form (99) (Box 3).

Box 3 **Nutrition and meta-organismal symbiosis.**

*The commensality of a superorganism*: Human native microbial population is constituted by 10–100 trillion bacteria that reside in the gastrointestinal tract (100). Their joint genome, defined as microbiome, has been estimated to include ≥100 times as many genes as the 2.85 billion base pair human genome (100), and it is able to perform tasks that humans have not evolved on their own (101). In this perspective, human beings should be reconsidered as “superorganisms” in co-evolution with their own microbiota, thereby possessing a “metagenome” (102). The gut microbiota is composed by different bacterial strains and species with considerable interpersonal variation, which evolved to exert a strong influence on the human metabolic phenotype.

Accordingly, the gastrointestinal IS evolved the capability to distinguish between harmful pathogens and harmless symbiotic (commensal) microorganisms, generating a strong effector response toward the former and remaining unresponsive to the latter. The gut bacteria can be roughly divided into three classes on the basis of the intensity of the host response in intestinal epithelial cells (IECs) and dendritic cells (DCs): (i) pathogenic microorganisms, which are virulent and induce a strong host response; (ii) probiotics, which modulate certain IEC and DC functions and induce an intermediate response; (iii) and commensal bacteria, which exhibit homeostatic control of the immune response (103). The reaction of gastrointestinal IS toward gut microbiota is in part triggered by signaling interactions of the surface molecules of probiotic bacteria (such as long surface appendages, polysaccharides, and lipoteichoic acids) with host PRRs and by bacterial cell surface macromolecules, which are key factors in this beneficial microorganism–host crosstalk (103).

*Immune system and microbiota: crosstalk, modulation, and cohabitation*: The modes of action by which probiotics are thought to contribute to human health fall into three main categories. First, certain probiotics can exclude or inhibit pathogens (through competition/cooperation for nutrients, antimicrobial production, competitive exclusion, cell–cell communication processes explicated by means of adhesions, lactic acid, bacteriocins quorum-sensing signals, etc.). A second mechanism enhances the function of the intestinal epithelial barrier by modulating the various signaling pathways that lead, for example, to the induction of mucus and defensin production, the enhancement of cell tight junction functioning, and the prevention of apoptosis. The third mechanism modulates the host immune responses, by regulating the cytokine expression, with local and systemic effects on phagocytosis, modulation/induction of DCs and different pro- and anti-inflammatory T cell subsets (Th1, Th17, and T_reg_ lymphocytes).

Intriguingly, gut microbiota showed to modulate the IS not only in the gastrointestinal tract but also in other human compartments like the central nervous system with potential implication in autoimmune diseases such as multiple sclerosis (104). Tolerance and cohabitation between microbiota and IS are owed to an incredibly complex, balanced network of interaction and cross-influences among elements of the two systems, and to a highly dynamic maintenance of very special environment and interface.

Indeed, mucosal surfaces are the largest contact area of the body with its environment. The human intestinal mucosa alone is 100-fold larger than the skin (105). Moreover, this specialized surface is covered by a single layer of epithelial cells with absorptive properties. In this perspective, the gut mucosa can be viewed as an interface rather than a barrier between the “inside” and “outside.” Environment-derived materials find in the enterocytes and the specialized epithelial cell called M cells of the gut act as a selective but permeable gate of entrance into the body.

Lymphocytes that compose the gut-associated lymphoid tissue (GALT) are either part of lympho-node-like structures such as Peyer’s patches or scattered throughout the lamina propria and intraepithelial spaces of the intestine in such a way that it is impossible to distinguish functionally epithelia and lymphoid components. B-cell-deficient mice, for instance, have a defect in the formation of M cell (106), thereby suggesting that B lymphocytes are not only lodged there but also provide signaling molecules for the differentiation of the gut epithelia.

There are two major consequences that follow the contact between these antigens and the gut lymphoid tissue: the production of secretory immunoglobulin (Ig) A and the induction of oral tolerance. The secretory IgA (SIgA) is a non-inflammatory subclass of immunoglobulin that is present in all mucosal secretions and can remove pathogenic microorganisms without inflammatory responses (107). Oral tolerance is a phenomenon known in medical literature since 1909 when Besredka showed that guinea pigs fed with milk-containing chow could not be immunized against milk proteins (108). In this scenario, tolerance means the suppression of inflammatory immune responses to the fed antigen and it has a close resemblance to the tolerance toward self components. Since this first description until very recently, oral tolerance has been only marginally quoted, probably due to the fact that a tolerance acquired to non-self food antigens represent a hard challenge to the SNS paradigm. In the 1970s oral tolerance was the topic of some systematic and important studies since suppressor T cells were proposed to explain the inhibitory effect generated by feeding antigens (108, 109). With the recent revival of T_reg_ cells and their important role in maintaining central as well as peripheral tolerance, the suppression induced by oral administration of antigens came back into the scene, being the oral route a very efficient way to induce peripheral tolerance both in animal models (110) and in humans (111). Oral tolerance probably accounts for the robust balance that keeps the homeostasis of intestinal mucosa with its highly activated lymphoid tissue (108). We are all tolerant to the food proteins that we ingest and also to our microbiota, as documented in mice and humans (111–113).

This hyporresponsiveness achieved by feeding, however, does not imply antigenic ignorance; it rather depends on active immune recognition and non-inflammatory responses. In fact, the mechanisms involved in oral tolerance seem to be similar to the ones triggered during central tolerance in the thymus, by inducing anergy/deletion of specific T cells and of T_reg_ cells (108). Indeed, CD4+ T cells can be converted into CD25+ activated forkhead box P3 (FoxP3+) regulatory cells in the intestinal mucosa by the action of CD103+ specialized dendritic cells (DCs) that secrete retinoic acid (114, 115). Other regulatory cells able to secrete transforming growth factor-β (TGF-β) are also generated in the intestine and these cells are involved in the gut homeostasis and in the induction of tolerance to antigens that reach the body through the gut (114). Therefore, the GALT has thymus-like mechanisms that are able to treat the antigens coming from the diet and from the autochthonous microbiota as if they were self antigens. The similarities between oral tolerance and natural tolerance was the inspiration for a large number of successful studies showing that autoimmune disease models can be either prevented or treated by oral administration of self components (110).

It has been already shown that the state of tolerance to self as well as oral tolerance to fed antigens is concomitant to a highly immune activated state (116, 117). Indeed auto-reactive antibodies as well as auto-reactive T lymphocytes can be found in healthy individuals. Autoantibodies of the IgM, IgG, and IgA classes, reactive to a variety of self components such as serum proteins, cell surface structures, and intracellular structures are “naturally” found in all normal individuals (118). These natural antibodies are produced mainly by B1 cells and were shown to be polyreactive (119, 120). SIgA is also known to be produced by B1 as well B2 cells in the gut lamina própria. Gut B1 cells secrete IgA in a T-cell independent fashion and these antibodies are highly polyreactive (121, 122). Interestingly, Quan and coworkers (123) have also shown that SIgA found in the intestinal lumen could be the secretory counterpart of the natural antibodies in serum. Significant levels of SIgA antibodies to human actin, myosin, tubulin, and spectrin have been detected in saliva and colostrum samples from normal subjects. In addition, a recent study shows strikingly that antibiotic-induced changes in gut microbiota have strong influence on the TCR repertoire of host T_reg_ cells selected in the thymus, and not on induced T_reg_ cells (124).

Another interesting aspect of the immune reactivity initiated at the mucosal sites is that these natural antigens have a critical role in the development of the IS itself. Since weaning up to adulthood, mice that are fed with a diet where antigenic-intact proteins are replaced by amino acids bear an immature IS and a poorly developed GALT. They showed decreased levels of SIgA and, more remarkably, they had lower levels of serum IgG and IgA, and a profile of cytokine production biased toward T helper 2 (Th2) resembling neonates (98). Similar alterations were described in adult germ-free mice (125), indicating that the removal of antigens from diet or microbiota hampers the regular development of the IS.

Therefore, if we think about nutrition as a process implying a continuous construction and shaping of the immune self, then dietary antigens and autochthones microbiota, actively participating in food digestion, represent exactly a blurry, liquid zone where foreign materials become autocomponents and are treated as such by the IS.

The human gut-associated lymphoid tissue keeps the intestinal microbiota under control by a “constitutive low-grade physiological inflammation,” which is grounded on a net of positive and negative feedback processes. The particular biological architecture of the gastrointestinal mucosal IS allows the distinction between harming pathogens and symbiotic microorganisms, causing a robust effector reaction toward the former and remaining tolerant to the latter. However, non-infectious human diseases characterized by an abnormal intestinal inflammation, such as inflammatory bowel diseases, metabolic syndrome, allergies, as well as genetic defects in enterocytes PRRs system, can trigger a failure of the homeostatic equilibrium at the interface between the intestinal microbiota and the host. In this perspective, it has been suggested that the inflammatory process could be triggered and fostered by an aberrantly activated immune response to the constituents of the gut microbiota, which may be due either to a reduced mucosal tolerance, or to the age-related alterations in the gut microbiota composition, or to both (126). Nutritional deficit and age-associated tissue fault and injuries may also contribute to trigger a pathogenic inflammatory response in the presence of normally harmless symbiotic bacteria (127).

Studies (128) provided a deeper outlook of the association between the gut microbiota composition and the levels of numerous serum inflammatory markers. A reorganization in the population of butyrate producers and other bacteria with anti-inflammatory properties was observed in a model of an extremely aged and consequently compromised microbiota. The subsequent dysbiosis may be among the origins – or the consequences – of the increased proliferation of opportunistic enterobacteria, which appeared to be positively correlated to an increase in certain pro-inflammatory signals (IL-6 and IL-8). The authors conjectured that the age-related increase of pathobionts can either contribute to inflammaging, or be fostered by the systemic inflammatory status.

Finally, human beings have been recently considered as “metaorganisms” as a result of an intimate symbiotic liaison with the intestinal microbiota (100, 129). This postulation forces a more holistic vision of the aging process, where dynamics of the interaction among environment, intestinal microbiota, and host must be taken into consideration. Starting from birth, a dynamic microbial ecosystem develops from a sterile environment and colonizes the gut ecosystem (130). Later on, the developmental modifications in the gut mucosa and in the intestinal IS, and the introduction of a solid diet drive the shift to a resilient adult-like profile of the human gut microbiota, characterized by a significant microbial biodiversity. The aging of the gut microbiota begins after a subject-specific “threshold age,” which depends on individual features such as diet, environment, country, and eventually, frailty. In any case, alterations of nutrition regimen, lifestyle, and the immunosenescence of the intestinal IS dramatically impact the microbial ecology of the human GI tract, and conversely, the manipulation of the gut microbiota may result in modification of the functionality of an aged IS (131).

## Molecular Mimicry and Proteasome-Catalyzed Peptide Splicing: When Self and Non-Self Coincide

Molecular mimicry phenomena are suggested to be among the causes of pathogenic autoimmunity. Molecular mimicry is supported by the homology of human-derived sequences and pathogen proteome, as well as by the phenomenon of T cell receptor (TCR) degeneracy (85), whereby epitopes with different sequence are recognized by the same TCR (132). It is known that the TCR is able to operate at the level of the single receptor (affinity of the TCR-ligand bond) as well as at the emerging level that derives from integration of multiple signals by the collective of interacting cells (concentration of TCR ligands and related number of bound TCRs) (82, 133). This condition determines a continuum of inputs to the TCR (“TCR signalosome”) determining the various cell functional outcomes (133), which is difficult to relate to a rigid concept of ligand structure and of self.

So far, TCR degeneracy seems to play a stronger role for MHC class II-restricted than I-restricted epitopes, and therefore it appears to be more relevant for the activity of CD4+ T cells (134) as demonstrated in some autoimmune diseases such as multiple sclerosis (135).

Viruses, bacteria, and human proteome share a limited number of 9-mer peptide sequences. Such a number is higher between bacteria and human proteome than between virus and human proteome, although the degree of overlap between bacterial and human proteomes does not correlate with bacterial pathogenicity (136, 137). Such observation is relevant because the epitopes that are bound to MHC class I molecules and presented to CD8^+^ T cells are generally 9-mers. These epitopes are usually generated by proteasomes, which by their selective cleavage preferences, determine which epitope sequence will represent human or pathogen antigens on the surface of APCs. The proteasome is the central catalytic unit of the ubiquitin proteasome system, which is responsible of the non-lysosomial degradation of the 80–90% of proteome (138). A small part of the produced peptides is transported to the ER, bound by MHC class I molecules and presented at the cell surface to CD8^+^ T lymphocytes for the immune recognition. This antigen presentation pathway is usually restricted to the proteasome-dependent processing of self- and viral-proteins because of the cellular compartmentalization of the antigens. Proteasomes take also part to the presentation of bacterial antigens as well as of cytoplasmic antigens loaded to the MHC class II (139, 140). Therefore, the proteasome generates the majority of peptides that label self and non-self antigens for the T cell-mediated inflammation. Antigen presentation is generally increased after interferon-γ (IFN-γ) stimuli because it induces, amongst the others, the synthesis of alternative proteasome catalytic subunits and the concomitant formation of immuno-proteasomes (141). Standard- and immuno-proteasomes differ in their preferential cleavage of peptide sequences (Mishto et al., personal communication). However, they generate same MHC class I epitope repertoires although in different amount (Mishto et al., personal communication). Such quantitative differences could explain the qualitative differences in epitope repertoires generated by the two proteasome isoforms described in some studies (142–145).

Often, immunoproteasome has been shown to better generate MHC class I-restricted viral epitopes, whereas several self epitopes (including tumor epitopes) have been described to be better generated by standard proteasome (146–148). Such apparent dichotomy between viral and human proteome epitopes is not due to discrimination at proteolytic level between self and non-self by proteasome isoforms. On the contrary, it caused by the fact that the identification of MHC class I-restricted epitopes often depends on the *ex vivo* isolation from the peripheral blood of CD8^+^ T cells. These cells escaped the negative selection in the thymus, because they did not efficiently recognize self MHC class I-restricted epitopes presented by thymic APCs. The latter cells express mainly immunoproteasome. Consequently, in the thymus the presentation by APCs of those epitopes that are better produced by immunoproteasome leads to a depletion of the reactive thymocytes. On the contrary, the thymocytes that react against epitopes better generated by standard proteasome could maturate, migrate outside the thymus, and become potentially auto-reactive and anti-tumoral cytotoxic T lymphocytes (CTLs). In summary, although standard proteasome do not generally better produce self epitopes, the absence of this proteasome isoform in the thymus could permit the survival of CD8^+^ T cells that recognize self epitopes, including tumor-associated epitopes that are better generated by standard proteasomes. Because viral epitopes are not presented in the thymus, such a discrimination toward CD8^+^ T cells specific for epitope preferentially generated by immunoproteasome does not occur (149).

The different expression of proteasome isoforms in thymus (mainly thymus- and immuno-proteasomes), in dendritic cells (immunoproteasome), and in parenchyma cells (mainly standard proteasome) (150) influences, from another angle, the definition of SNS. Indeed, the same human proteome-derived antigen is represented by quantitative different repertoire of epitopes exposed onto the MHC class I molecules according to the proteasome isoforms present in the cells that is exposing the epitopes. Therefore, a variation of the proteasome population as occurs upon inflammation or chronic pathologies may lead to the recognition of human antigen as non-self (151). One more time, it is important to bear in mind that such alteration of the MHC class I-restricted epitope repertoire upon alteration of the proteasome population are only quantitative.

Among the tumor-associated MHC class I-restricted epitopes, it is worthy to mention also epitopes produced by proteasome-catalyzed peptide splicing (PCPS). Indeed, proteasomes produce peptides during the degradation of proteins by a simple hydrolysis or by PCPS. PCPS is a cut and paste reaction, which can occur also by binding two peptides derived from distinct polypeptides (*trans* PCPS) (152, 153). PCPS has been demonstrated *in vivo* only for four MHC class I-restricted epitopes (152, 154–157) although a sizeable number of proteasome-generated splicing peptides has been identified by digesting *in vitro* different polypeptides (153, 158). Intriguingly, the proteasome-generated splicing peptides are more prone than normal cleavage products to be potential MHC class I-restricted epitopes because of structural characteristics of PCPS catalytic sites (158).

These recent inputs could impact on the definition of SNS from at least two different points of view. First, PCPS increases the possible number of epitopes generated from human or pathogen proteomes (159) thereby increasing the chances of molecular mimicry. Second, PCPS might be theoretically generated by the ligation of peptides derived from two distinct proteins (*trans* PCPS) (152, 153, 158). In case both human and viral proteins had been processed by *trans* PCPS, we might observe the formation of chimeric epitopes, half human half virus. In our opinion the likelihood that such chimera epitopes are produced in normal conditions is extremely low. However, during an acute viral infection, where infected cells are forced to synthesize viral protein in high amount, the chances of a PCPS event between human and viral peptides might significantly increase becoming something more than a purely academic speculation.

## Age-Associated *N*-Glycans Modifications and Inflammation: When the Aged Self Becomes Non-Self

Glycosylation is a form of co-translational and post-translational modification that attaches glycans to proteins, lipids, or other organic molecules, affecting protein folding and stability as well as influencing their biological activity. Glycosylation patterns reliably reflect cellular phenotypic state and appearance of altered carbohydrate structures may constitute a pivotal phenotypic alteration that alarms the IS to danger and initiate a reaction. Recently, a model has been proposed that considers the cellular glycosylation status as a critical indicator of cellular health status, and that is being interpreted by the IS *via* the carbohydrate receptors involved in the regulation of effector cells (160). A tight linkage was identified between the concentration of *N*-glycans depleted of galactose residues and the aging process, progeroid syndrome, and a variety of autoimmune and inflammatory diseases (160). The agalactosylated structures are prevalently found linked to the asparagine residue 297 of IgG (IgG-G0), the main serum immunoglobulin. Indeed, the percentage of IgG-G0 tend to increase with age having the lowest concentration at the age of 25 years (161–163). Recently, studies performed with high-throughput technologies confirmed the association of agalactosylated structures with *chronological* age and reported for the first time that the age dependent hypogalactosylation is not restricted to the IgG fraction of serum antibody, but is common with the other serum glycoproteins. A study on healthy centenarians in comparison with people aged 60–90 years reported that in the above-90 years cohort, the increase of the concentration of agalactosylated structures and the concomitant decrease of digalactosylated structures (the so-called Gly co-age test) characterize also the extreme elderly (164, 165).

The IgG-G0 serum concentration is not only a putative marker of chronological age, but also of *biological* age. Indeed, a study based on the Leiden longevity study, a longevity model based on nonagenarian sibling pairs, offspring and their partners as control, revealed that the IgG-G0 concentration was lower in the offspring cohort than in the partners’ ones, indicating that the offspring cohort seem “less aged” that the age matched controls (166). Moreover, a clear link between the increase of IgG-G0 and altered inflammatory pathways was identified and several mechanisms of pro-inflammatory action were described. The IgG-G0 possesses a five-fold complement activation activity than other IgG. This enhanced activity is due to the high affinity that IgG-G0 has with mannose-binding lectin that activates complement through the lectin pathway (167, 168). Moreover, IgG-G0 interacts with lectin receptors, such as the mannose-binding receptor and DC-SIGN, of macrophages and dendritic cells (DCs), thereby increasing the lectin uptake sustaining the inflammatory process (169, 170).

Taken together these observations strongly suggest a link between the IgG-G0 imbalance and inflammation. In this perspective, the simple IgG-G0 accumulation observed in age is a transformation of physiological pattern into a pathological one (altered carbohydrate structures), or in other words of self to non-self that triggers inflammatory responses (171).

In a recent study about the role of epigenetics in human aging and longevity, one of us observed an age-related drop in global DNA methylation and a delay of this process in centenarians’ offspring (172). Remarkably, literature data suggest a relationship between the drop of DNA methylation detected during aging and the occurrence of age-associated diseases (173–176). Genome-wide methylation analyses evidenced DNA methylation profiles specific for aging and longevity, showing genes involved in nucleotide biosynthesis, metabolism, and control of signal transmission are differently methylated in centenarians’ offspring (vs non-centenarians’ offspring) hypothesizing a role for these genes in human longevity. Such results suggested that a better preservation of DNA methylation status, a slower cell growing/metabolism, and a better control in signal transmission through epigenetic mechanisms may be involved in the process of human longevity. In particular, the identification of a relevant number of age-related hypomethylated loci significantly enriched of genes associated with inflammatory response suggests a role of epigenetics in the modulation of inflammatory processes in aging (172).

## Testable Predictions and Falsifiability

To validate our hypothesis, we need to demonstrate that the various parameters of immunological biography (procedural, timing, space, geography, etc.) alter the self during lifetime. To conceive a test, we hypothetically need genetically identical organisms that the more they age in different conditions (reared separately), the more differently they will react to the same antigenic stimulus. About the contribution of environmental factors in shaping the immune self, monozygotic (MZ) twins seem to us the best model. Far more complex is to figure out the type of the immune stimulus and its quantitation. Thus, we have focused our attention on a series of well-established experiments in MZ twins as possible model to test the liquid self hypothesis.

- Studies in twin vaccinology demonstrate that genetics controls the early phase of the vaccine antibody response, whereas environmental determinants predominantly influence antibody persistence and avidity maturation (177). The role of timing and the consequential long-term effect of a single immune insult can be also evaluated in unrelated individuals. In 12-month infants, for instance, avidity and isotype maturation of measles vaccine-induced antibody are affected by age, proving insight into ontogeny of the immune response to measles vaccine (178).
- Accumulated evidences support the notion that environmental factors can have a long-term effect on epigenetic profiles and influences the susceptibility to disease in MZ twins, which at certain age shows a variable degree of discordance with respect to different features. In relation with autoimmune diseases, the identification of DNA methylation changes, DNA methyltransferases and histone modification enzymes in individuals who develop various autoimmune diseases, are attracting the attention of researchers in the epigenetics field (179, 180).
- In identical MZ twins that underwent kidney transplant, the intra-uterine effects, epigenetic differences, differential antenatal environmental factors as well as age may cause a recipient to develop antibodies to minor or non-HLA antigens, which may impact graft survival (181).
- Human CMV is a common herpes virus establishing lifelong persisting infection, which has been implicated in immunosenescence and mortality in the elderly. Evidences from the Leiden longevity study and the longitudinal study of aging Danish twins suggest that susceptibility to CMV infection, even under continuous within-partnership exposure, appears to be more strongly influenced by early-life environment than by genetic factors and adult environment (182).
- Differences between the members of MZ twins possibly related to environmental and “immune-biographical” differences can also be found in other reports. For example, Tazi and coworkers (183) showed that MZ twins infected at birth from the same blood transfusion contaminated with HIV-1 had not only very different clinical outcomes (twin A relatively healthy and with slower disease progression compared to twin B) but also phylogenetic differences, higher growth rates and higher genetic diversity in the HIV population.
- Variable incidence rates in invasive disease and vaccine performance among different populations are also important to support the idea that IS is molded and shaped by evolutionary forces. As an example, the incidence of invasive *Haemophilus influenzae* type b (Hib) disease among the Navajo and White Mountain Apache children is 20 times greater than that observed among coeval children in the general US population, despite the general decline of the disease after the implementation of Hib vaccine (184). Similarly, relatively high Hib carriage rates are observed in Alaskan natives despite high rates of vaccine coverage (185). Moreover, efficacy of the same Hib conjugate vaccine is different in Finland and Alaska (186). Although such phenomena may be caused by both environmental and genetic factors, different antibody response to Hib vaccine is also present among twins (46), revealing the link between proximate immunological experiences at the level of individual’s lifetime with long-term, distant, evolutionary selective force operating at the level of population.

Given these premises, we surmise that a testable prediction of the liquid self hypothesis should be designed as follows: two (groups of) genetically identical inbred mice should be reared in two different “clean” and “dirty” environments (in terms of quality and quantity of feeding, immune insults, stress, microbial environment). The immune self of these two differently aged, reared apart mice should after a consistent time (e.g., 2 years) be changed in a significant way. Accordingly, they should respond in a different way to several different immune stimuli, regarding quantity and quality of the immune response, from low-grade stimuli to an “immunological storm” such as a transplant. In particular, in the case of an isograft between the two differently reared mice, the absence of any sign of rejection would disprove the validity of the liquid self theory. On the contrary, if the presence and the intensity (quantification) of the immune response, the length of the period before the possible rejection will be significantly different in comparison with a transplant between two mice of the same sex, strain, and litter but reared in the same environment and exposed for 2 years to the same diet and so on, then this would mean that the immunological identity varies significantly and that self is age- and geography-dependent.

Instead of the dualistic acceptance or rejection of grafts, or complete absence/presence of immune reaction to a vaccine, we can also consider a more realistic situation where consistent variations in immunological parameters (antibody concentration, avidity, number of T regulatory cells, cytokine levels, among other) can occur as a consequence of a different immunological biography. We will consider this type of scenario in good agreement with our liquid self theory, even if we agree that this type of evidence is not a crucial demonstration. Indeed, immunological biography is expected to change the self along a continuum of states, in a liquid fashion, and does not forcedly predict a drastic overturning of the immune self.

## Conclusion and Perspective: Toward a New Conceptualization of Immunological Identity

At present the danger/damage theory is the most comprehensive theory on immunological self and represented a breakthrough in the discipline by reorienting the response of the IS toward entities that do alert (danger) and cause a damage rather than toward those that are foreign.

However, the danger theory and its recent refinements, being focused on the characteristics of the molecules capable of triggering an immune response, did not pay enough attention to the responding host. Indeed, organisms capable of an immune response belong to different populations or strains characterized by different genetics, different immunological experience related to lifestyle, exposure to specific pathogens, among others (75). Within this perspective, nutrition has a particular importance, owing to its interaction with the gut microbiota. Moreover, a major variable in immune responses is represented by age, overarching the 9 months of intra-uterine life till the extreme ages (centenarians).

Accordingly, our hypothesis of liquid self suggests that the immunological self can change and be modulated by the global immunological experience of individuals, i.e., by what we have suggested to call “immunological biography.” The concept of “immunological biography” synthesizes the individual immunological history, and has the capacity to take into account both the *qualitative* as well as the *quantitative* and the *temporal* aspects of the immunological stimuli that each of us – as an *accumulator* and *elaborator* of antigenic stimuli (42) – is exposed to in our lifelong history (Table 1).

**Table 1:** **Central tenets of the liquid self hypothesis**.

∙	The immunological self is dynamic, because it varies continuously depending on the sum of host’s immunological experiences and ecological context
∙	Time, i.e., evolutionary (population) and individual (intra-uterine, post-natal, adult, and extreme age) history, and environmental related factors (geographical location, nutrition, and lifestyle), something that we collectively name “immunological biography,” mold the immune identity, changing what the immune system will react to
∙	The immunological self is continuous, and not simply binary (i.e., self/non-self), and it reconfigures along a continuum of states
∙	The sum of these characteristics (dynamicity, timing, continuity) shapes the self in a liquid fashion

Indeed, immunity and immune mechanisms emerge (and evolve at the individual as well as the population level) in parallel with physiological processes such as nutrition and aging, which can be considered integral component of “normal” physiology. A variety of data in the literature suggest that early immunological stimuli *in utero* and during early infancy (40, 187), as well all the other stimuli impinging on the IS lifelong (including all the subclinical and persistent infections) can re-direct the IS toward different functional capabilities, thus predisposing the single individuals to different diseases, including those age-related.

Thus, our hypothesis of liquid self represents an extension of the danger/damage theory being focused on the responding hosts and their spectrum. We surmise that our hypothesis complements the danger/damage theory by adding a temporal and spatial dimension, which until now remained neglected and not properly conceptualized (25).

Another set of phenomena such as para-flammation (188), meta-flammation (189), and inflammaging (190) can be easily integrated in the “liquid self” hypothesis. The latter would recapitulate and represent the (interdisciplinary) point of convergence of such types of sterile inflammation. In particular, the low-grade systemic inflamed aging phenotype (inflammaging) is maintained by cell autonomous mechanisms (the aged micro- and macro-environment) and can propagate from cells to cells and from organs to organs, so that local and systemic inflammatory stimuli sustain and reinforce each other in a complex circuitry.

Similar phenomena are well known in the cancer field where the propagation of damaged DNA, DNA damage response, and inflammation to bystander cells has been conceptualized as para-flammation (191).

**Figure 1:**
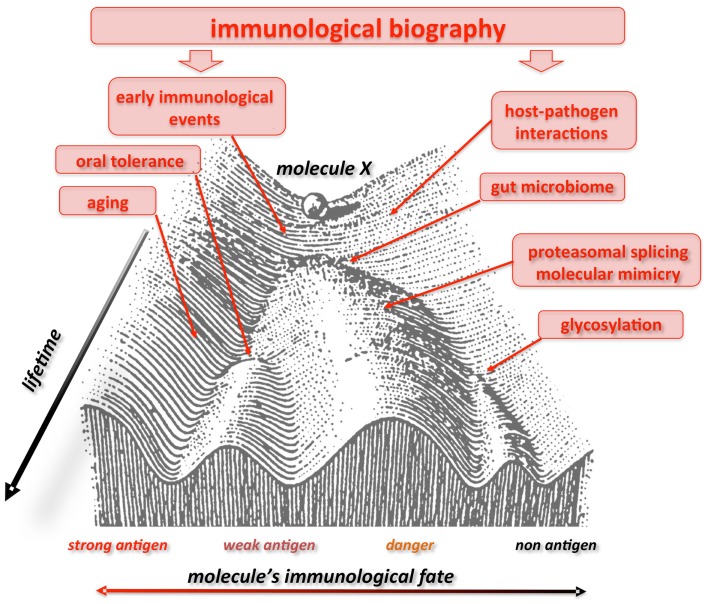
**Waddington landscape of self/non-self**. The immunological fate of a molecule or a molecular motif is not firmly fixed. Here this process is depicted in the context of the epigenetic landscape proposed by C. Waddington in 1940. A given molecule X “differentiates” into various “immunological profiles” (with more or less immunogenicity), like a ball rolling down the slope of a valley. Immunological experiences during lifetime push the molecule up and down the slopes and valleys, and, according to age, geography, and context (valleys, bifurcations, attractors), it can be perceived by the immune system as a strong, weak or non-antigen, a danger signal, an allergene, etc, i.e., showing different and changing degrees of immuno- genicity all through the individual’s course of life. Consequently, what today could elicit an immunogenic response, tomorrow would not. Figure modified from Ref. (192), originally presented in Waddington, C. H. Organisers & genes. 1940, Cambridge: Cambridge University Press.

Finally, our liquid self hypothesis allows to arrange the recent discontinuity theory in a wider, systemic framework (48) and offers a variety of testable predictions. Along these lines, to consider a single, defined molecule self or non-self, without considering its complex context (responding host), can be misleading. We predict that a given molecule could indeed be “seen” by the IS like autologous, antigen, allergene, auto-antigen depending on the “immunological landscape” carved by specific selective pressure acting on the individual’s history. Waddington advanced the “epigenetic landscape” during the 40s to visualize the various developmental pathways a cell might take toward differentiation (192). Similarly, the immunological landscape could offer a vivid representation of how fluctuating environmental force during lifetime may alter the evolutionarily fixed genetic response to an immune stimulus (193) (Figure 1). Furthermore, both the epigenetic and immunological landscape stress the dynamic view of ontogenetic (biographical) selective pressure represented by multidimensional aspects of time, space, and context (valleys, bifurcations, attractors).

## Conflict of Interest Statement

The authors declare that the research was conducted in the absence of any commercial or financial relationships that could be construed as a potential conflict of interest.

## Abbreviations

DAMP, damage-associated molecular patterns; DCs, dendritic cells; ER, endoplasmic reticulum; FoxP3, forkhead box P3; GALT, gut-associated lymphoid tissue; IS, immune system; Ig, immunoglobulin; IFN, interferon; MHC, major histocompatibility complex; PAMP, pathogen-associated molecular patterns; PCPS, proteasome-catalyzed peptide splicing; T_reg_, regulatory T cells; SNS, self/non-self; TCR, T cell-antigen receptor; Th, T helper cells; TGF-β, transforming growth factor-β.
